# Engineering protein prenylation: an emerging tool for selective protein modification

**DOI:** 10.1042/BST20253076

**Published:** 2025-08-28

**Authors:** Sneha Venkatachalapathy, Caitlin Lichtenfels, Carston R. Wagner, Mark D. Distefano

**Affiliations:** 1 Department of Chemistry, University of Minnesota, Minneapolis, MN, 55455, U.S.A; 2 Department of Medicinal Chemistry, University of Minnesota, Minneapolis, MN, 55455, U.S.A

**Keywords:** biocatalysis, bioconjugation, enzymatic protein modification, farnesylation, farnesyltransferase, site-specific labeling

## Abstract

Prenyltransferases catalyze the attachment of isoprenoids to cysteine residues located near the C-termini of proteins including those containing a ‘CaaX’ tetrapeptide motif. This enzyme family includes farnesyl transferase (FTase), geranylgeranyltransferase type I (GGTase I), and GGTase type II (GGTase II). The CaaX motif broadly consists of cysteine (C), two aliphatic residues (a), and a variable residue (X), which determines substrate specificity for farnesylation and type I geranylgeranylation. This review primarily focuses on FTase-mediated protein modification strategies for assembling therapeutically valuable proteins. First, the process of protein prenylation and the structural features of the FTase active site are discussed. This is followed by an exploration of FTase-catalyzed bioconjugation of monomeric proteins and peptides, emphasizing its efficiency, modularity, and potential for industrial biological applications. The broader applicability of this approach is then highlighted in the design and assembly of multimeric protein structures, facilitating the development of complex biomolecular architectures with enhanced functionality, stability, and therapeutic potential. Finally, FTase mutagenesis strategies are examined that expand substrate scope, accommodating diverse functional groups for a wide range of biotechnological and therapeutic applications.

## Introduction

Bioconjugate therapeutics are macromolecular drugs designed by attaching therapeutic agents to biomacromolecules using covalent chemical linkers. A typical bioconjugate consists of three key components: 1. macromolecules, such as polymers, lipids, peptides, or proteins; 2. therapeutic agents, which can include small molecules or macromolecular drugs; and 3. covalent chemical linkers that connect the components [1]. The conjugation of chemical payloads to biomolecules to achieve selective delivery is a well-established approach, with 13 FDA-approved antibody-drug conjugates (ADCs) on the market as of 2024 [2]. Over the last two decades, it has expanded to include small peptide or antibody-derived protein binders with radioactive payloads [3], multimeric proteins [4,5], RNA molecules [6] with targeting groups, and modifications to the surfaces of large protein complexes [7]. While the diversity of targets, biomolecules, and payloads has rapidly evolved, the bioconjugation chemistry used in most clinical candidates has remained relatively constant. Traditional reactions, such as maleimide–cysteine or N-hydroxysuccinimide ester–lysine coupling, are still commonly employed. These methods are reliable and yield high conversion rates, but they have notable drawbacks. First, proteins must contain reactive residues on their surfaces, which limits the versatility of the approach. Second, the lack of regiospecificity in these reactions, as seen with lysine being a common surface residue, often leads to heterogeneous products with varying degrees of modification, making precise control challenging. In contrast, cysteine, while less abundant, is often modified with maleimides. However, the resulting thiosuccinimide bond formed between cysteine and maleimide is unstable and susceptible to decomposition, which can cause premature linker cleavage and reduce the overall stability of the conjugate [8–10]. Finally, the harsh conditions required for these reactions can destabilize proteins, and the use of excess of electrophilic reagents is often necessary to drive reaction conversion. While advancements in small molecule conjugation methods, such as next generation maleimides [11], have addressed some of these issues, there remains a strong demand for mild, catalytic methods to produce chemically defined bioconjugates.

Enzymes can play a key role in bioconjugation. For protein modification, they offer significant advantages over traditional chemical methods, including speed, regio-, and stereoselectivity, and the ability to operate under mild conditions, preserving biomolecular integrity [12]. In the last 20 years, a wide variety of enzymes that promote different types of reactions have been explored for this purpose, demonstrating their potential for creating precisely defined protein conjugates [13–15]. Those include sortase A [16], microbial transglutaminase [17], biotin ligase [18], formylglycine-generating enzyme [19], lipoic acid ligase [20], and farnesyl transferase (FTase) that have been employed for site-selective protein modifications in diverse biological applications. However, only a few review articles have examined FTase-mediated bioconjugation [14,15,21,22], and none have focused exclusively on this subject. Recently, our laboratory and others have leveraged the high specificity of that enzyme to site-specifically modify peptides and proteins. This review highlights the versatility of this approach and recent advancements, focusing particularly on the diverse biomolecule bioconjugates produced using this enzyme.

## Protein prenylation: a promising approach for enzymatic protein labeling

**Figure 1:**
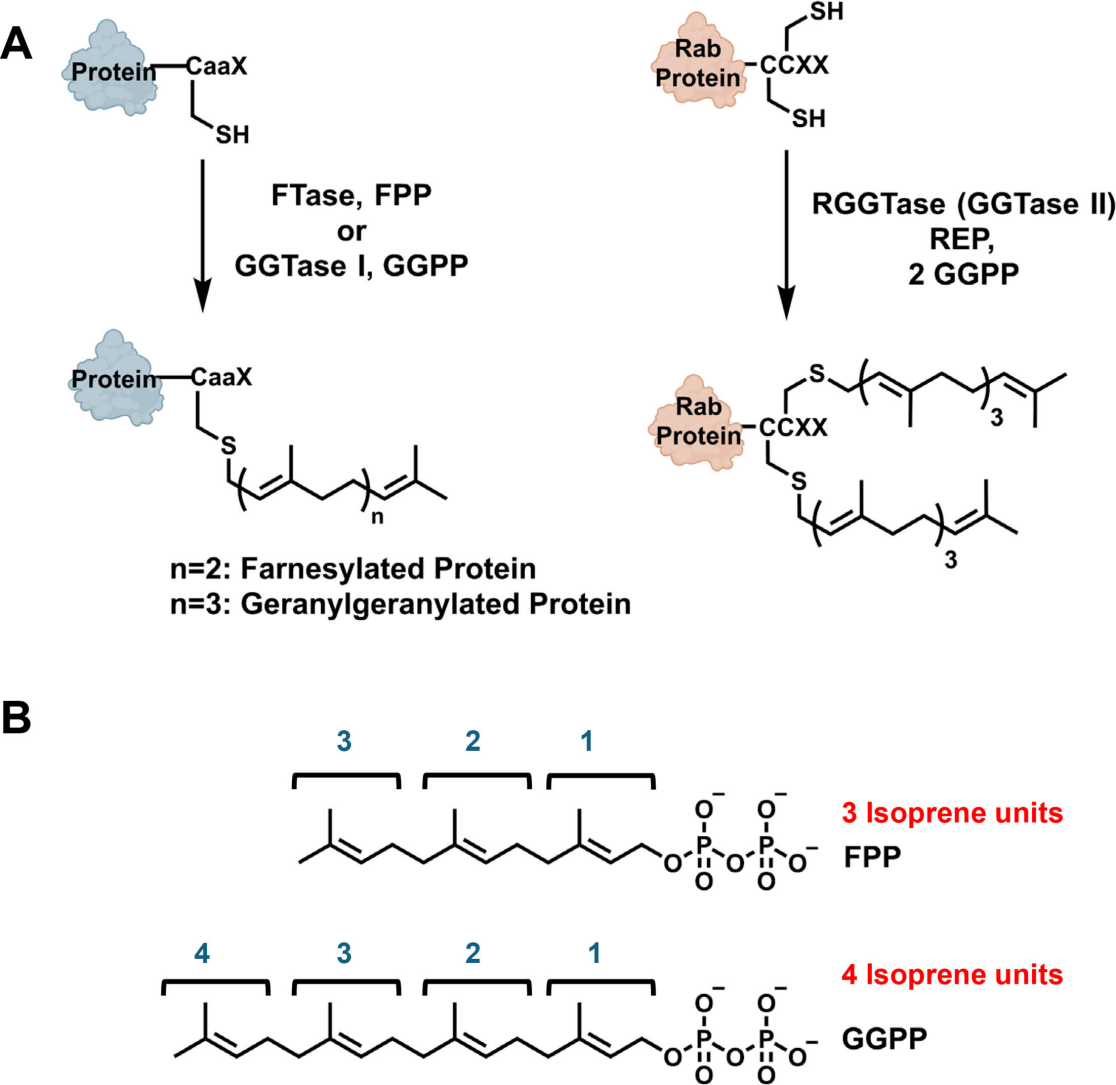
Overview of protein prenylation and its natural substrates. (**A**) Schematic overview of protein prenylation. (**B**) Structures of natural prenylation substrates. Farnesyl diphosphate (FPP) consists of three isoprene units, while geranylgeranyl diphosphate (GGPP) contains four.

Protein prenylation is a post-translational modification in which isoprenoid groups are covalently attached to proteins, playing a crucial role in membrane localization and protein–protein interactions [23,24]. This modification regulates mechanosensitive cellular processes, including migration, proliferation, and metabolism, by modulating target proteins and cytoskeletal dynamics [25]. This process is catalyzed by three main enzymes including FTase, geranylgeranyltransferase type I (GGTase I), and Rab GGTase (GGTase II). FTase and GGTase I modify a wide range of proteins, while Rab GGTase specifically targets the Rab subfamily of G proteins. A fourth enzyme, GGTase III, has recently been identified, but its substrate scope is currently limited to two proteins [26,27]. The prenyltransferases FTase and GGTase I transfer farnesyl or geranylgeranyl groups from their respective diphosphates, farnesyl diphosphate (FPP) and geranylgeranyl diphosphate (GGPP), to a cysteine residue positioned within a C-terminal ‘CaaX’ motif via a thioether bond (Figure 1A). FPP contains three isoprene units, while GGPP contains four isoprene units (Figure 1B). The adjacent aliphatic residues (‘a’) and the identity of ‘X’ are key determinants of substrate specificity, directing modification by either FTase or GGTase I. Specifically, methionine, serine, glutamine, or alanine at the ‘X’ position favors FTase recognition, while leucine or phenylalanine directs modification by GGTase I [28–30]. For FTase and GGTase I, modification requires only a four-residue C-terminal motif and proteins lacking such a natural ‘CaaX’ sequence can be genetically engineered to include one, enabling the modification of virtually any protein with bioorthogonal functionality. In contrast, substrate recognition by GGTase II is more complex and less useful for protein labeling applications [31]. FTase (and GGTase I, to some extent) displays exceptional substrate specificity and flexibility, tolerating various isoprenoid analogs containing bioorthogonal groups including azides, alkynes, aldehydes, ketones, and strained alkenes. These functionalized isoprenoids enable site-specific protein labeling, drug delivery, and visualization, expanding the utility of protein prenylation for biotechnological and therapeutic applications [28].

## Active site and mechanism of FTase

**Figure 2:**
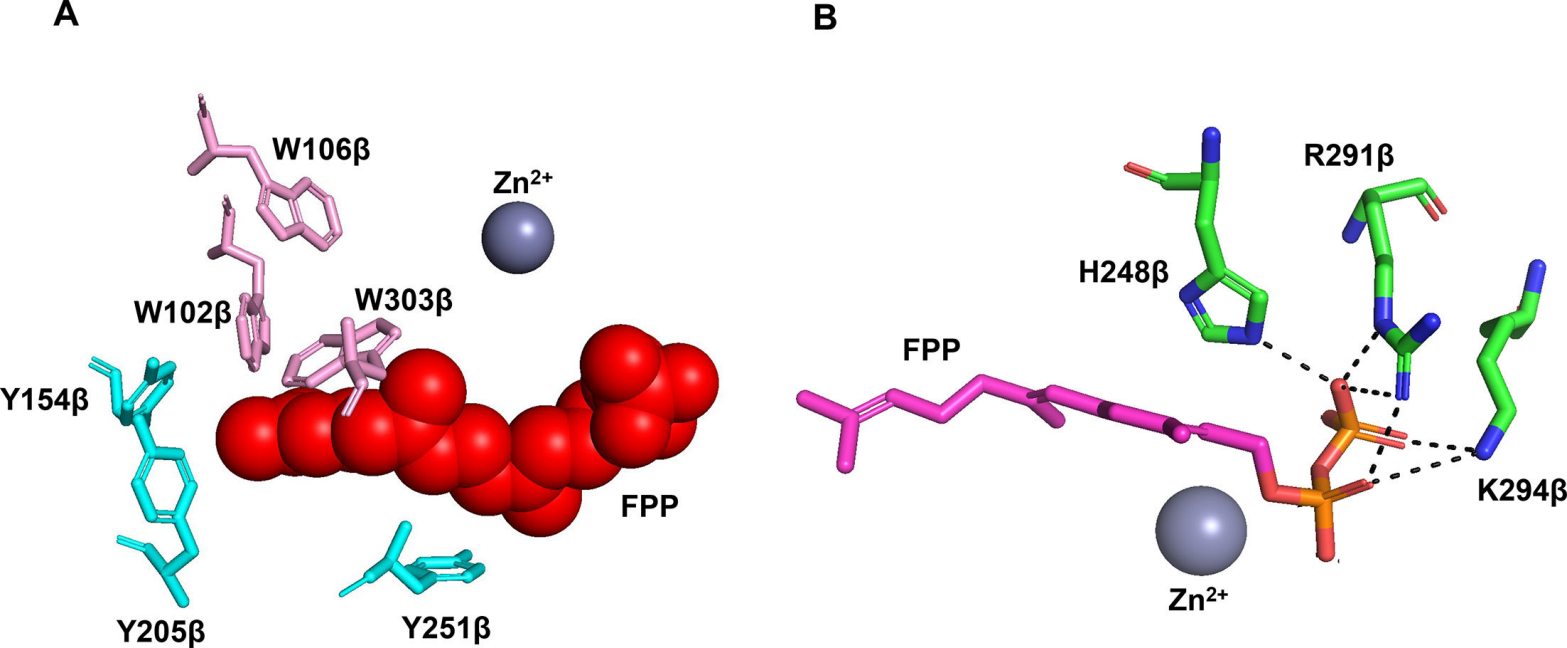
The FPP binding pocket of FTase (PDB: 1JCR). (**A**) The hydrophobic cavity formed by tyrosine (cyan) and tryptophan (pink) residues, with FPP bound shown in red and Zn²^+^ in gray. (**B**) Polar interactions between the diphosphate group of FPP (pink sticks) and histidine, arginine, and lysine residues. Zn²^+^ is shown in gray. FPP, farnesyl diphosphate; FTase, farnesyltransferase.

X-ray crystallographic analysis reveals that FTase is a heterodimer composed of 48 kDa α and 46 kDa β subunits. It requires a zinc ion for catalytic activity, which enhances CaaX peptide binding and increases thiol reactivity via formation of a Zn-thiolate species. The β subunit adopts an α-α barrel fold with six parallel helices at its core and six peripheral helices. The catalytic zinc ion is positioned at the top of the barrel near the subunit interface, defining the active site. A deep, funnel-shaped hydrophobic cavity lined with conserved aromatic residues provides a binding site for the farnesyl group of FPP. The cysteine residue in the CaaX motif is positioned adjacent to the FPP a phosphate, facilitating farnesylation. Highly conserved aromatic residues Trp 303β, Tyr 251β, Trp 102β, Tyr 205β, and Tyr 200β form hydrophobic interactions with the isoprenoid (Figure 2A), while the diphosphate moiety of FPP binds in a positively charged cleft near the subunit interface (Figure 2B), adjacent to the catalytic zinc ion. Zinc ligands include Cys 299β, Asp 297β, and His 362β [32], while the diphosphate moiety forms hydrogen bonds with His 248β, Arg 291β, Lys 294β, and Tyr 300β. The depth of the hydrophobic cavity dictates isoprenoid substrate specificity by discriminating between different chain lengths [33–36]. The stabilization of CaaX substrate binding is facilitated by interactions with side chains from both the α and β subunits, forming hydrogen bonds with Lys164α, Arg291β, and Lys294β [33]. The hydrogen bond between the CaaX substrate C-terminus and Lys164α is particularly crucial, as mutating this residue abolishes FTase activity. The removal of the positive charge at Lys164α significantly affects the association rate constant and binding affinity of a CaaX peptide substrate, suggesting its essential role in stabilizing the enzyme–FPP–peptide ternary complex [37]. The allylic diphosphate structure present in FPP is necessary for effective turnover. A variety of mechanistic experiments including stereochemical investigations [38,39], kinetic isotope effect measurements [40–42], steady-state [43,44], and single-turnover kinetic analysis [45,46] suggest that this results from the reaction proceeding via an associative mechanism with some cationic character.

## Bioconjugation of monomeric proteins via farnesyltransferase: advances in biotechnological and therapeutic applications

Bioorthogonal chemistry enables rapid, selective, and mild reactions in biologically compatible environments without disrupting native processes. The integration of bioorthogonal chemistry with enzymatic labeling has revolutionized chemical biology, particularly in site-specific functionalization of therapeutically useful molecules [13,15]. This section explores bioconjugation strategies using engineered synthetic isoprenoid substrates for FTase (Table 1), enabling the incorporation of bioorthogonal handles into proteins. These modifications facilitate click chemistry-based functionalization, expanding the scope of biomolecule assembly for diverse biotechnological and therapeutic applications.

**Table 1:** Steady-state kinetic parameters for FTase-catalyzed prenylation with FPP and engineered synthetic substrates.

Substrate	*k*_cat_ (s^−1^)	*K*_*M*_ (µM)	*k*_cat_/*K*_*M*_ (s^−1^ µM^−1^)	Rel *k*_cat_/*K*_*M*_	Ref.
FPP	1.3	1.7	0.77	1	[47]
C10-Azi C15-Azi C20-Azi C10-DHAzi	0.45	2.1	0.21	0.27	[47]
C15-DHAzi C5-Alk C10-Alk C15-Alk	1.1	0.67	1.6	2.1	[47]
C15-Ald	0.13	1.9	0.07	0.10	[48]
C10-BA	0.015	1.0	0.01	0.01	[49]
C10-BK C11-K	0.0028	2.6	0.001	0.001	[49]
C10-TCO	0.10	0.77	0.13	0.16	[50]
C10-Nor	4.6	0.88	5.2	6.8	[50]
C10-BAK	0.012	2.5	0.005	0.01	[49]

### Staudinger ligation

**Figure 3:**
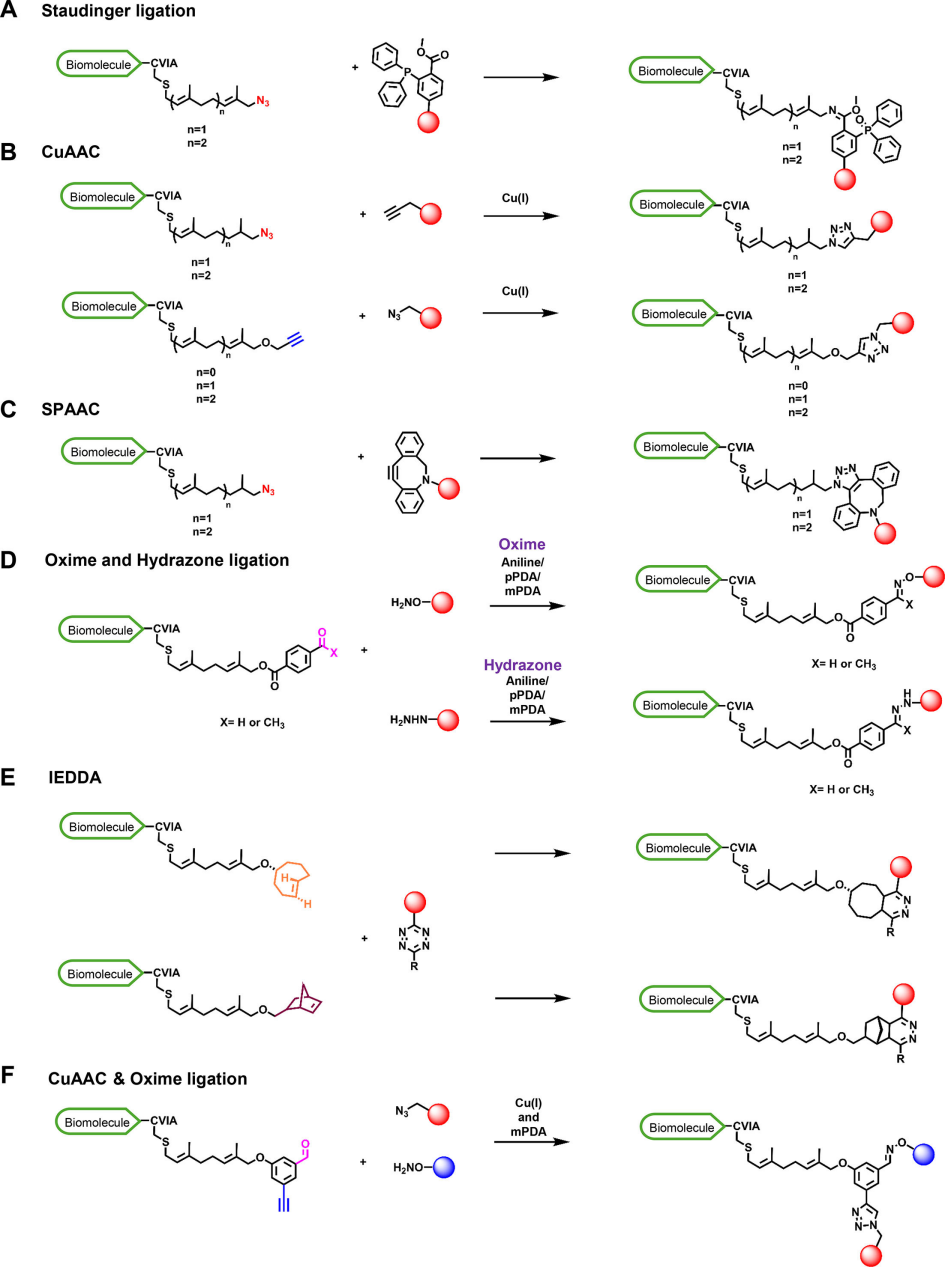
Schematic representation of various bioorthogonal reactions on biomolecules (peptides/proteins) bearing a C-terminal CVIA CaaX motif. These proteins are enzymatically labeled by FTase with isoprenoid analogs containing bioorthogonal handles. The red and blue spheres represent either reporter groups (e.g., fluorophores, cytotoxins, PEG chains, DNA) or surfaces (e.g., agarose beads, gold surfaces, glass slides). FTase, farnesyltransferase; PEG, polyethylene glycol.

**Figure 4:**
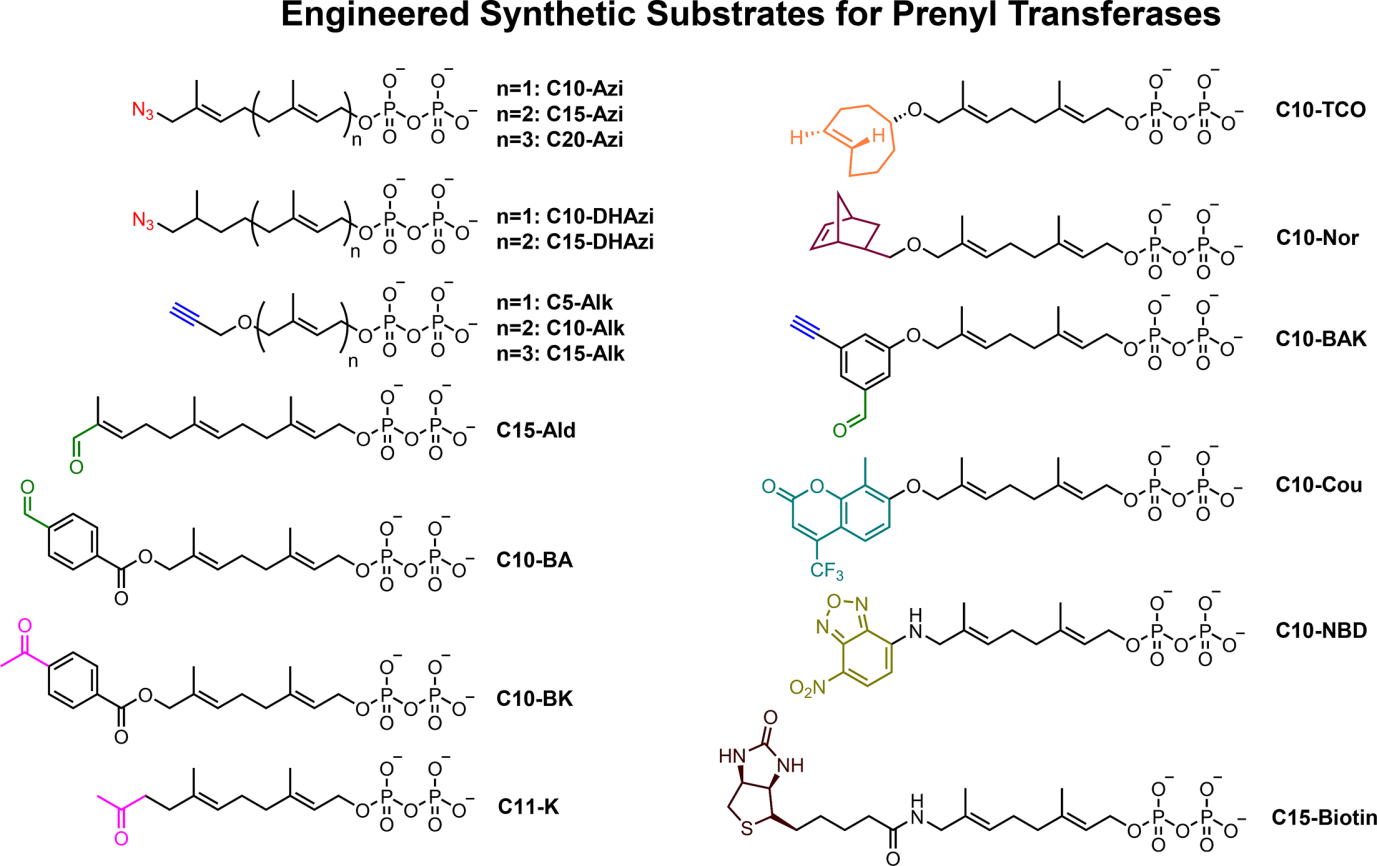
Chemical structures of engineered synthetic substrates for prenyl transferases, featuring diverse bioorthogonal handles at the terminus for further modification through various bioorthogonal chemistry reactions. Color coding: azide (red), alkyne (blue), aldehyde (green), ketone (ketone), trans-cyclooctene (TCO, orange), norbornene (magenta), coumarin (cyan), nitrobenzoxadiazole (NBD, yellow ochre), and biotin (brown).

Azides are a versatile functionality for click chemistry due to their small size and stability, making them truly bioorthogonal since they are absent in virtually all biological systems. The Staudinger ligation between an azide and a functionalized triphenylphosphine was the earliest bioorthogonal click reaction developed specifically for biological purposes (Figure 3A). This classic reaction continues to be widely utilized in a variety of contexts including cellular environments and live animals, owing to its exceptional selectivity and biocompatibility [51,52]. The Staudinger reaction outcomes differ significantly between aryl and alkyl azides. Aryl azides often form stable imidate linkages, whereas alkyl azides yield amide products due to differences in stability, nucleophilicity, and steric effects [53,54]. Site-specific incorporation of compound **C10-Azi** (Figure 4) an allyl azide, into a CaaX peptide via FTase followed by Staudinger ligation resulted exclusively in an O-alkyl imidate-linked product, similar to those observed with aryl azides, rather than the expected amide-linked product. This modified Staudinger product demonstrated remarkable stability, maintaining its integrity even under acidic conditions for 24 hours, highlighting its potential for diverse applications [47,55]. Likewise, compound **C15-Azi** (Figure 4) was efficiently incorporated into peptides by FTase with comparable efficiency [47,56]. Aryl azide-containing isoprenoid analogs have also been evaluated as FTase substrates for Staudinger ligation [47]. Overall, the use of Staudinger ligation with prenyl azides enables precise single-site protein modification of CaaX motifs that is useful for site-specific protein immobilization on glass slides [57]. Furthermore, the efficient synthesis of **C10-Azi** makes it particularly suitable for large-scale applications, while the strategic incorporation of azide functionalities on proteins expands the scope of bioorthogonal modifications for advanced biotechnological and therapeutic applications.

### Copper-catalyzed azide alkyne cycloaddition

Slow reaction kinetics (*k* ∼ 10^–3^ M^–1^ s^–1^) and oxidation-prone phosphine reagents [54] have prompted the exploration of alternative click chemistries, leading to the development of the copper-catalyzed azide alkyne cycloaddition (CuAAC), the most widely used click chemistry today. CuAAC takes advantage of a Cu(I) catalyst that accelerates the formation of the triazole heterocycle (Figure 3B), resulting in significantly enhanced kinetics (*k* ∼ 10–100 M^–1^ s^–1^) [58–60]. The dihydro azide analogs **C10-DHAzi** and **C15-DHAzi** (Figure 4), which lack the terminal alkene, have been identified as FTase substrates with broad applications. These analogs are particularly advantageous for avoiding isomerism issues arising from sigmatropic interconversion that occurs with allylic azides [61,62]. Once incorporated into the protein of interest, the azide functionality allows for CuAAC with virtually any alkyne functionalized reporter group or material.

#### Förster resonance energy transfer applications

One valuable application of fluorophore incorporation into proteins via FTase is Förster resonance energy transfer (FRET), which enables the study of molecular interactions and structural dynamics. To facilitate this, alkyne substrate analogs **C5-, C10-, and C15-Alk** (Figure 4) with varying isoprene units, ranging from one to three, were developed and validated as effective FTase substrates using a combination of kinetic assays, HPLC, and mass spectrometric characterization [63–65]. Results indicate that **C15-Alk** effectively labeled both GGTase I and FTase peptide substrates, suggesting its potential as a dual enzyme substrate for prenyl transferases [66]. Subsequently, an alkyne-functionalized Enhanced Green Fluorescent Protein (eGFP) was covalently coupled to a Texas Red fluorophore carrying an azide moiety. FRET analysis measured a Förster distance of 40 Å between the eGFP chromophore and Texas Red, closely matching predictions from molecular modeling [63]. This orthogonal labeling strategy complements cysteine-based modifications, allowing for dual-labeling approaches in FRET studies to investigate large protein assemblies with enhanced precision and flexibility.

#### Surface immobilization

Site-specific, covalent attachment of biomolecules to surfaces with precise spatial control is critical for studying protein-protein [67], protein-DNA [68], and protein–small molecule interactions [69], as well as for array technologies and diagnostic applications [70–73]. The CuAAC reaction is a robust method, forming stable triazole linkages while maintaining protein activity. This scalable approach is suitable for large-scale applications and works with both purified proteins and complex mixtures [74]. Several studies have explored FTase-mediated protein immobilization. For example, eGFP was successfully immobilized on alkyne-containing agarose beads [61] and alkyne-coated glass slides [57] via CuAAC. Minimizing non-specific labeling is essential for efficient protein capture in biotechnological applications. FTase-mediated installation of an alkyne or azide on eGFP allowed analysis of immobilization efficiency on agarose beads, where azide-functionalized beads exhibited 12% less non-specific uptake of unmodified eGFP after 18 hours. These findings suggest that alkyne-functionalized proteins, when paired with excess azide reagents, offer more selective, site-specific incorporation with reduced background signals [63,75]. The hydrophobic nature of isoprenoids can affect the stability of modified proteins. To address this, a notable study examined the effect of a shorter isoprenoid substrate. The **C5-Alk** alkyne substrate containing a single isoprene unit (Figure 4) was found to reduce hydrophobicity during protein modification, improving stability. Additionally, carboxypeptidase Y was used to enzymatically remove the final three amino acids of the CaaX-box, essential for prenylation, without affecting the prenylated cysteine. To compare the effects of substrate size on protein stability, homohexameric hemolysin-coregulated protein 1 (Hcp1) from *Pseudomonas aeruginosa* was used with shorter (**C5-Alk**) and larger (**C15-Alk**) alkyne substrates. High-speed centrifugation and Western blot analysis revealed that C5-modified Hcp1 remained soluble, while C15-modified Hcp1 precipitated, highlighting the stabilizing effect of the shorter substrate [64]. This approach allows precise modification of proteins and peptides with alkyne functionality, adding a single cysteine modified by only nine non-hydrogen atoms. Alkynes offer greater stability than azides, particularly aryl azides, which are susceptible to reduction by thiol-based reducing agents such as dithiols (e.g., dithiothreitol) [76] and monothiols (e.g., β-mercaptoethanol and glutathione) [77]. This enhanced stability further expands their utility in biomolecular research.

Chemoselective immobilization is also vital for micro- and nanofabrication, which underpins innovations in biomedical devices and nanotechnology by enabling the creation of chemically defined structures [78,79]. Arrays with antibodies oriented in a uniform, regioselective manner exhibit significantly higher sensitivity than those with random orientations, offering up to 100-fold enhancement in assay sensitivity. Recombinant antibody-binding proteins (A, G, and L), produced in E. coli and chemoselectively functionalized with alkynes via FTase-catalyzed reaction in cell-free homogenates, were directly attached to azide-stamped glass surfaces without purification. The covalent immobilization of truncated versions of these proteins in a well-defined orientation via a regioselective chemoenzymatic approach via CuAAC enabled the effective binding of a wide range of antibodies. An attractive feature of this method is that bound antibodies could be stripped from the surface, extending the slide’s useful life and permitting its reuse with different antibodies and ligands [80]. Similarly, FTase-mediated enzymatic functionalization of mCherry, GFP, and GSTase (glutathione S-transferase) with **C15-Alk** provided a platform for selective, covalent protein stamping on azide monolayers assembled on gold surfaces through click reactions, demonstrating its potential for controlled surface modifications [65,81]. Immobilization has improved enzyme properties for biotechnological applications in environmental and medical sectors [73]. FTase-mediated regioselective modification of GSTase with bioorthogonal isoprenoid appendages enabled covalent immobilization on glass slides via CuAAC. A GSTase activity assay, performed using glass wells immobilized with the modified enzyme, confirmed high activity. GSTase immobilization was carried out using both cell-free homogenates and purified proteins. Covalently immobilized GSTase retained its catalytic activity, suggesting preservation of its native structure. However, non-specific immobilization was observed, likely due to competition with other proteins in the cell-free homogenate for reactive sites on the glass surface. This issue was mitigated by using purified azide- or alkyne-functionalized GSTase-CVIA proteins for immobilization [74].

#### DNA–protein cross-links

DNA–protein cross-links (DPCs) are large, damaging lesions caused by antitumor drugs, toxins, radiation, and free radicals [82]. Investigation of biological effects and repair of DPCs requires the synthesis of suitable DNA substrates [83]. While general chemical methods can be used to prepare such molecules, those procedures typically lead to heterogeneous mixtures, clouding the interpretation of subsequent data [84]. Hence, access to structurally defined DPCs is highly desirable. Two notable studies showed that structurally defined DNA−protein [85] and DNA−peptide conjugates [86], generated using CuAAC between C8-alkyne-dU in DNA and FTase tagged azide proteins or peptides, can be obtained in high purity using biorthogonal chemistry. Polymerase bypass experiments with DPC substrates showed that while larger DPCs blocked all human translesion polymerases tested, polymerases η, κ, and ι bypassed a smaller DPC prepared from a decapeptide. That study suggested that large DPCs in cells may require proteolytic processing to be effectively repaired and that large intact DPCs are mutagenic, highlighting the potential of FTase enzyme-mediated site-selective modification to investigate structurally complex DPCs.

### Strain-promoted azide alkyne cycloaddition

While the CuAAC reaction is rapid and highly efficient, the use of Cu(I) or Cu(II) creates a number of problems in biological experiments. In addition to its general toxicity, Cu salts induce protein oxidation and DNA strand breaks, limiting the utility of CuAAC for molecular studies of living systems. Additionally, the CuAAC reaction often requires optimization of metal, ligand, and auxiliary reductant concentrations [87]. Strain-promoted azide alkyne cycloaddition (SPAAC), which uses alkynes activated with ring strain to eliminate the need for metal catalysts, offers a safer, copper-free alternative for *in cellulo* and *in vivo* protein conjugation (Figure 3C) [88]. The SPAAC reaction is a powerful tool for constructing bioconjugates, including protein–oligonucleotide conjugates (protein-ODN), while avoiding copper-induced DNA cleavage. Protein-ODN conjugates are widely used in biological and biotechnological applications, including protein encoding in multiplexed libraries [89], immobilization for protein arrays [90], target identification in diagnostics via PCR [91] or mass spectrometry [92], enhanced cellular delivery of antisense oligonucleotides [93], and spatial organization of proteins into multimeric assemblies [94,95]. Site-specific conjugation enhances efficiency, as demonstrated by enzymatic incorporation of prenyl azides **C10-DHAzi** and **C15-DHAzi** into proteins, followed by reaction with ODN-linked strained alkynes including dibenzocyclooctyne (DBCO). This method, validated with model proteins and polypeptide hormones (eGFP, mCherry, HIV nucleocapsid protein, and glucose-dependent insulinotropic polypeptide [GIP]), functioned across a broad pH range (3.5–12.3), accommodating proteins with diverse isoelectric points. Additionally, SPAAC enabled the immobilization of azidoprenylated proteins onto ODN-functionalized beads under conditions similar to CuAAC discussed above [96]. This Cu-free method provides a viable alternative for synthesizing protein–DNA conjugates; however, its efficiency is notably reduced when protein availability is limited. It does not match Cu-catalyzed reactions in terms of yield and reaction rate.

### Oxime ligation and hydrazine ligation

The rate of SPAAC (*k* ∼ 0.1^–1^ M^–1^ s^–1^) is significantly slower than CuAAC, and the large size and hydrophobicity of ring-fused strained alkynes not only reduce their aqueous solubility but can also alter target molecule properties or cause off-target binding [97]. To overcome these limitations, imine-based bioconjugation has been explored as a promising alternative, with oximes being the most stable imines (Figure 3D) [98,99]. Although oxime ligation is inherently slow under physiological conditions, the development of amine-based catalysts has greatly enhanced its efficiency and utility (*k* ∼ 1–10^3^ M^–1^ s^–1^). This copper-free labeling strategy is highly effective for live-cell and *in vivo* PET imaging applications [100], with proven stability and efficiency. Its orthogonality to the Cu(I)-catalyzed click reaction enables precise, multisite protein modifications using distinct bioorthogonal chemistries. The development of **C15-Ald**, **C10-BA**, and **C10-BK** carbonyl substrates (Figure 4) for FTase enables the site-specific installation of carbonyl functional groups at the C-terminus of proteins with a CaaX box. Hydrazone ligation is more efficient than oxime ligation at high protein concentrations (>50 μM) due to faster kinetics, while oxime ligation is preferred at low micromolar concentrations due to its higher equilibrium constant. Aldehyde-functionalized purified polypeptides, such as eGFP and GIP, have been shown to be site-specifically labeled in solution with an aminooxy fluorophore or polyethylene glycol (PEG) polymer using 100 mM aniline catalyst, achieving nearly 100% oxime formation in under 1 hour [48,101]. Similarly, aldehyde-functionalized protein immobilization via oxime or hydrazone bond formation with aminooxy or hydrazine tagged agarose beads has been established as an efficient copper-free alternative, valuable for various biotechnological applications. Unlike irreversible immobilization via oxime bonds, which proceeds with low efficiency (~30%), hydrazone-based immobilization (Figure 3D) is reversible through transoximization with functionalized alkoxyamines in the presence of an amine-based catalyst, resulting in high immobilization efficiency (~95%) in under 1 hour. This process enables the release of site-specifically modified proteins through oxime formation under mild, physiological conditions without denaturation. This ‘capture and release’ methodology is particularly useful for functionalizing impure proteins, as many protein labeling applications involve targets that are not pure or abundant, making specific modification strategies for crude mixtures essential [48,102]. Protein release kinetics and oxime formation efficiency are key to the success of the methodology. Although aniline accelerates the reaction, its solubility limit in water (100 mM) prevents its use at higher concentrations. Screening for improved catalysts led to the discovery of phenylene diamines, with meta-phenylene diamine (m-PDA) outperforming aniline due to its higher solubility and Schiff base basicity. m-PDA catalyzes the reaction approximately two-fold faster at equal concentrations and can be used at much higher concentrations, accelerating the reaction up to 15 times faster than aniline. At 500 mM, m-PDA increases the protein release rate by 10-fold, and at 750 mM, it achieves a 15-fold increase [103]. Both m-PDA and p-PDA catalyze oxime ligation with similar efficiency but have distinct limitations. Excess m-PDA favors Schiff base formation over oxime ligation, resulting in incomplete reactions. Hence, the [m-PDA]/[aminooxy reagent] ratio should not exceed 250 to maintain efficiency. In contrast, p-PDA allows higher concentration and faster reactions without this limitation but is highly susceptible to rapid oxidation in air, leading to reduced catalytic activity over time. Numerous next-generation catalysts have been developed for this reaction, and which one to use depends on the specific application and reaction conditions [49,103–108]. Overall, this approach enables protein enzymatic modification in crude mixtures, selective immobilization on hydrazide beads in the presence of other proteins, followed by selective and efficient labeling and release back into the solution with high yield in just a few hours. This streamlined strategy for polypeptide modification could be especially valuable for large-scale production of protein conjugates in therapeutic or industrial applications.

Targeted therapy with ADCs and related protein scaffolds has become a well-established approach over the past 15 years, significantly enhancing drug delivery to cancer cells through receptor-mediated internalization. The homogeneity and stability of protein–drug conjugates (PDCs) are critical for optimizing therapeutic efficacy and reducing off-target toxicity [109,110]. In this context, chemoenzymatic modification via FTase has proven to be an effective strategy for creating well-defined, therapeutically valuable proteins. Antibody-mimetic proteins, including repebodies and Designed Ankyrin Repeat Proteins (DARPins), are smaller than full-size antibodies, potentially offering better tumor penetration and high affinity for overexpressed cancer target receptors [111–114]. A ketone-containing FTase substrate, **C10-K** (Figure 4) linked to a repebody selected for its high affinity for the epidermal growth factor receptor (EGFR), was efficiently conjugated to aminooxylated monomethyl auristatin F (MMAF) via oxime ligation, producing stable, homogeneous repebody–drug conjugates for targeted therapy [115]. Similarly, DARPins targeting the epithelial cell adhesion molecule (EpCAM), a diagnostic marker for various cancers, were conjugated to the MMAE warhead using a capture-and-release strategy with the aryl aldehyde **C10-BA**, either from purified proteins or directly from crude *E. coli* lysate, for cancer cell killing [116]. Both repebody- and DARPin-based drug conjugates exhibited receptor-specific cytotoxicity (IC_50_ in the nanomolar range) while effectively minimizing off-target effects. Notably, this approach has been successfully translated into clinical studies. One ADC currently in clinical trials, NCT03944499, employs FTase-based chemoenzymatic bioconjugation with a ketone substrate **C10-K** (Figure 4), enabling the orthogonal coupling of two MMAF payloads to HER2-targeting trastuzumab via oxime ligation, resulting in a homogeneous and stable ADC [117,118].

### Inverse electron demand Diels–Alder reaction

Oxime ligation, which depends on aldehyde or ketone functional groups, is limited by potential oxidation of aldehydes to inactive acids or the lower reactivity inherent to ketones. The slow kinetics of the Staudinger ligation and SPAAC reactions also pose significant challenges for certain applications when the biorthogonal reaction is performed *in vivo* where only low target concentrations are present. Tetrazine ligation, a bioorthogonal reaction between an electron-deficient tetrazine (Tz) and a strained trans-cyclooctene (TCO) derivative, follows the inverse electron demand Diels–Alder (IEDDA) mechanism (Figure 3E). With N_2_ as the only by-product, a rate constant of up to 10⁷ M⁻¹s⁻¹, high specificity, biological inertness, and tunable kinetics, tetrazine ligation is the fastest known bioorthogonal reaction, making it ideal for *in vivo* applications [119] including pre-targeting approaches [120]. In that latter approach, TCO-functionalized targeting proteins, such as antibodies or antibody fragments, are first administered to localize at the disease site. Subsequently, a tetrazine reagent is injected, enabling an *in vivo* click reaction to either release a drug at the target site via a ‘click-to-release’ mechanism [121] or covalently link a radioisotope chelator complex for biomedical imaging [122–124]. Accordingly, the TCO isoprenoid analog **C10-TCO** (Figure 4) enabled site-specific incorporation of a TCO group at the C-terminus of peptides or proteins, which were then ligated with tetrazine-functionalized reagents [125]. While **C10-TCO** manifested complete ligation with a tetrazine in as little as 15 minutes, the TCO moiety is synthetically difficult to produce, and the large hydrophobic group is too large to be efficiently processed by the enzyme [50,125]. Other cyclic alkenes, such as cyclopropene (*k* = 4.5 × 10^2^ M^−1^ s^−1^) [126] or norbornene (*k* = 10 M^−1^ s^−1^) [127], manifest reactivities with tetrazine slower than TCO but have been shown to be effective for various *in cellulo* labeling applications. Norbornene analog **C10-Nor** was used to functionalize a DARPin protein, enabling IEDDA-mediated conjugation with a tetrazine functionalized TAMRA fluorophore for *in cellulo* imaging application (Figure 3E) [50]. This strategy is a promising approach for the construction of protein conjugates for targeted delivery of anticancer toxins or drugs and represents a useful alternative to the SPAAC reaction.

### Multiplexed bioorthogonal labeling

Modification of proteins with multiple functional groups can be useful to rapidly assemble complex protein conjugates and assemblies. Enzymatic incorporation of a substrate analog containing both alkyne and aldehyde groups enables the generation of proteins with two orthogonal reactive sites for site-specific and simultaneous modification. This approach can be used to enhance protein specificity, reactivity, potency, and pharmacokinetics. A unique triorthogonal substrate **C10-BAK** (Figure 4) developed with aldehyde and alkyne moieties enables selective protein functionalization with two distinct moieties, including a fluorophore and PEG polymer (Figure 3F). Fluorophores facilitate biophysical studies and cellular localization, while PEG enhances pharmacokinetics. Simultaneous labeling with azido TAMRA and aminooxy PEG (3 k) achieved more than 95% conversion, with oxime ligation completing within approximately one hour and the click reaction within 12 hours. This strategy enabled the efficient one-step installation of two modification sites on a protein with quantitative conversion [128]. In that work, the rapid oxidation of p-PDA catalyst was further accelerated by the Cu-catalyzed click reaction conditions, making m-PDA the preferred choice for simultaneous protein labeling under those conditions [49]. This methodology was applied to the biomedically important Ciliary Neurotropic Factor protein, a potential therapeutic agent for slowing retinal degeneration [128]. Beyond these proteins, this approach could be used to enhance the drug-to-antibody ratio (DAR) in the ADC field, to facilitate the construction of multimeric protein assemblies, and expand its applications in therapeutic development.

### Engineering protein assemblies

#### DNA nanostructures

**Figure 5:**
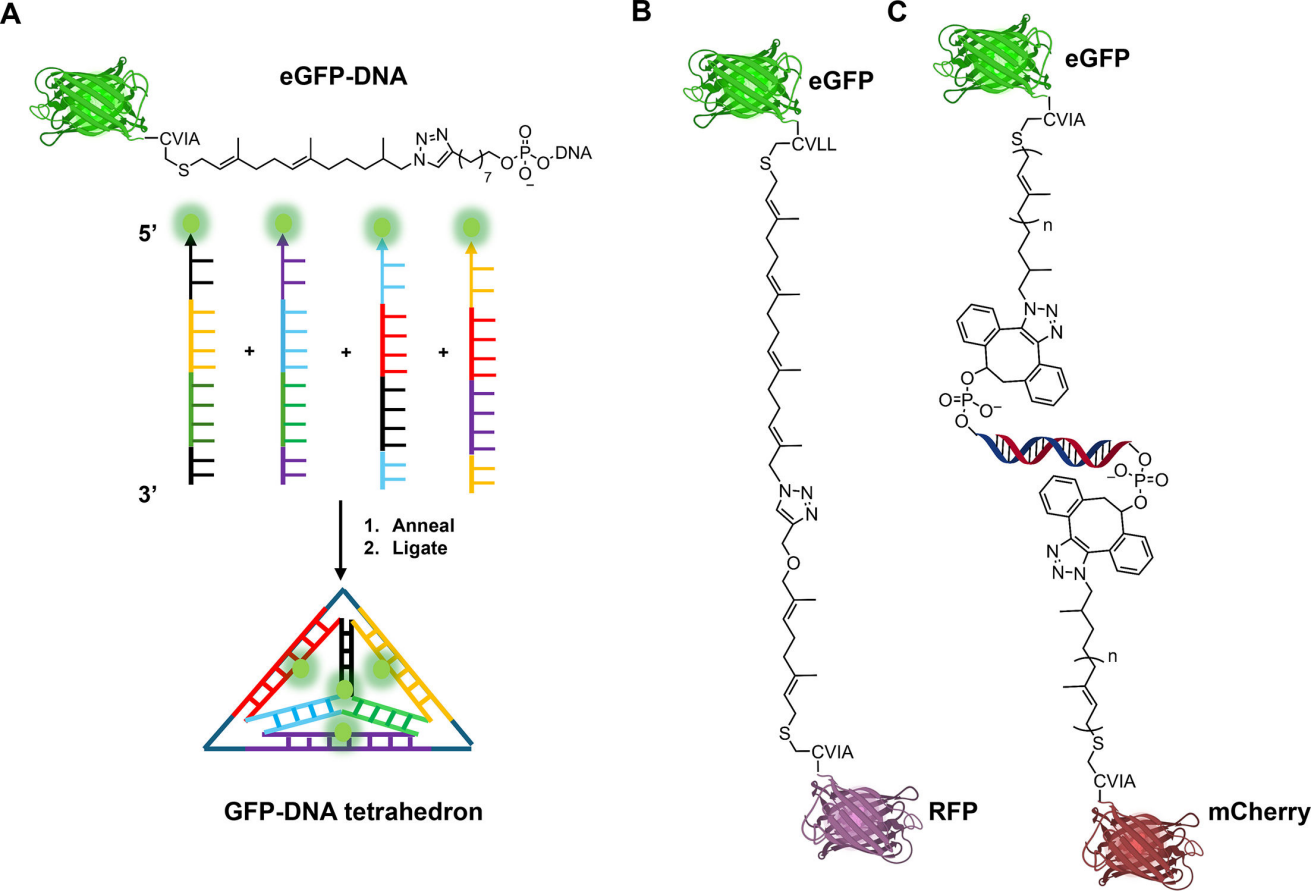
Structures of protein-DNA assemblies. (**A**) eGFP-DNA tetrahedron assembly. Four unique GFP-DNA constructs generated via CuAAC self-assembled to form a DNA nanostructure. (**B**) eGFP-RFP tail-to-tail heterodimer formed via CuAAC in a dual protein labeling fashion using rFTase and rGGTase I. (**C**) eGFP-mCherry heterodimer formation via DNA hybridization post SPAAC reaction to assemble protein-ODN conjugates (*n* = 1 or 2). eGFP, Enhanced Green Fluorescent Protein.

DNA serves as an excellent scaffold for nanostructure assembly due to its rigidity, predictable structure, and programmable hybridization, enabling the formation of nanoscale architectures including cubes, tetrahedra, and 2D arrays. Covalent DNA lipidation via CuAAC has enabled the integration of synthetic DNA nanopores into lipid bilayers by overcoming the repulsive energy barrier posed by the DNA backbone. Using this strategy, programmable nanopores capable of size-selective cargo translocation and stimulus-responsive gating have been created. These lipidated DNA nanopores hold potential for real-time sensing and other membrane-based applications [129]. Likewise, incorporating proteins into DNA nanostructures allows for the spatial organization of functional biomolecules, facilitating applications including immuno-PCR detection, sequential biocatalysis, and nanomaterial organization [95,130]. Protein–DNA conjugation methods include noncovalent approaches using streptavidin or aptamers and direct covalent modification utilizing cysteine or lysine residues. A regioselective chemoenzymatic strategy utilizing FTase enabled linking of a protein C-terminus with single-stranded DNA, allowing controlled protein assembly into nanoarchitectures via complementary hybridization. Using CuAAC, azide-functionalized GFP was conjugated to four distinct alkyne-functionalized DNA strands, generating four unique GFP–DNA conjugates. These conjugates were then incorporated into a self-assembling DNA tetrahedron using a strategy first developed by Tuberfield and coworkers [131]. Since the tetrahedron consists of four distinct DNA strands with unique sequences, it provides four individually addressable sites for protein functionalization. Single-molecule fluorescence measurements confirmed the programmed integration of up to four GFP molecules within the tetrahedral scaffold, demonstrating precise control over protein spacing and orientation in DNA-scaffolded assemblies (Figure 5A) [132].

### Protein homo- and hetero-dimers

Prenyltransferase-mediated protein labeling has been expanded by utilizing rat GGTase-I (rGGTase-I) and capitalizing on its different substrate specificity. By leveraging the distinct CaaX and isoprenoid substrate specificities of rPFTase and rGGTase-I, a simultaneous dual-labeling method was used to selectively modify two proteins, eGFP and RFP, using **C20-Azi** and **C10-Alk** (Figure 4), respectively. That one-pot reaction enabled efficient site-specific labeling and enabled the creation of tail-to-tail protein dimers via CuAAC (Figure 5B) [133]. This approach offers a flexible strategy for protein modification, with potential applications to *in vitro* heterodimerization and the construction of complex multidomain proteins.

Attaching ODNs to proteins enables the formation of supramolecular assemblies and surface immobilization via DNA hybridization. DNA-mediated protein assembly has been demonstrated using CuAAC-synthesized protein–DNA cross-links [85], and SPAAC-generated protein-ODN conjugates [96] (section IV. 2C and section V. A). Hybridization experiments with complementary DNA-linked GFP and mCherry proteins showed successful heterodimer formation, yielding a GFP-mCherry heterodimer (Figure 5C). However, SPAAC-generated protein–DNA conjugates required annealing at 55°C followed by cooling to 4°C, whereas CuAAC-generated conjugates hybridized efficiently at room temperature. This difference may stem from the increased hydrophobicity of the dibenzocyclooctyltriazole (DBTrz) linker in SPAAC conjugates, potentially hindering hybridization at room temperature. Nonetheless, this work highlights the potential of Cu-free bioorthogonal reactions as an effective strategy for assembling DNA-templated multimeric protein structures while maintaining protein functionality [96].

#### Chemically self-assembled nanorings

**Figure 6:**
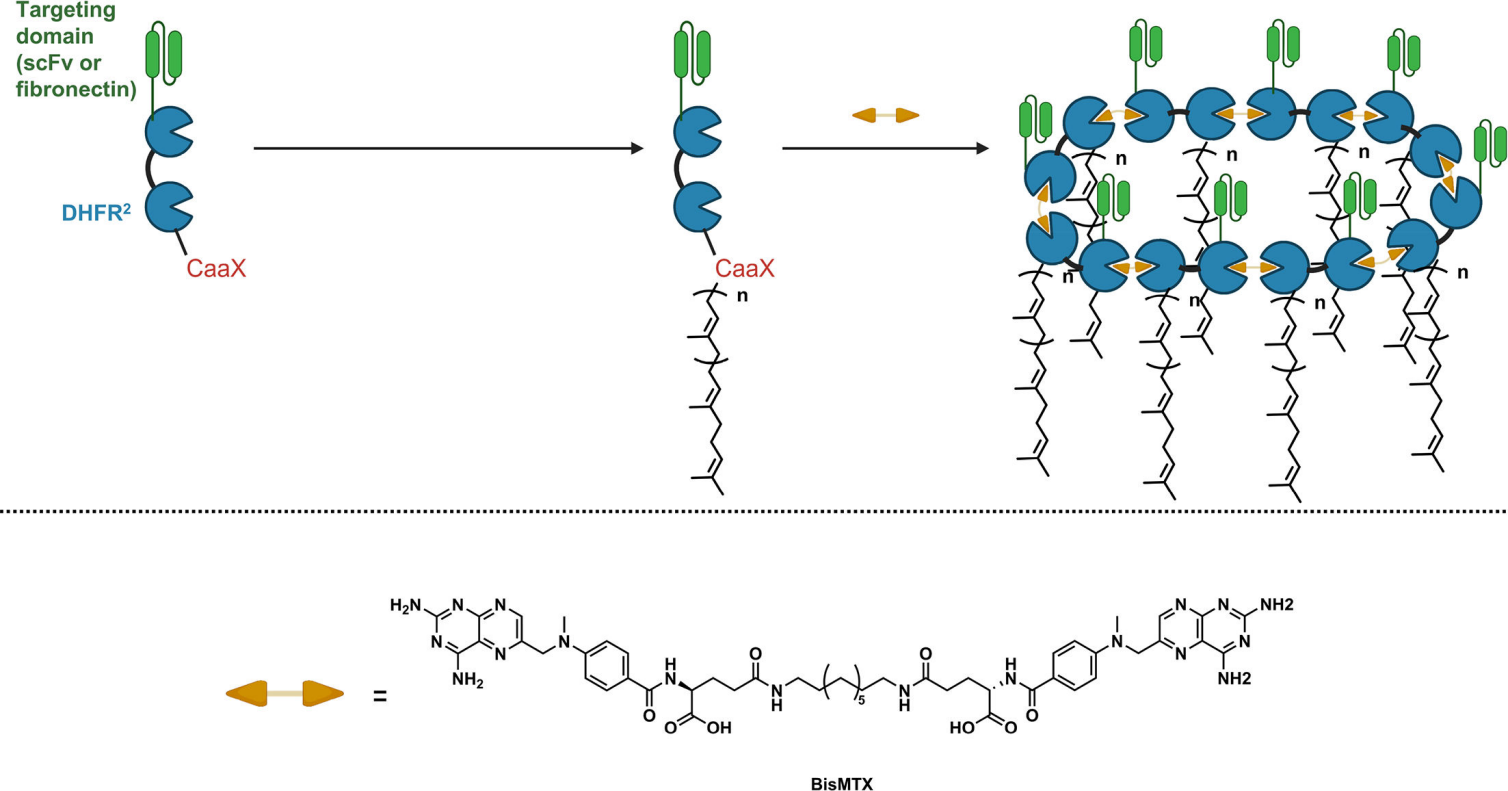
Enzymatic modification of the DHFR^2^ backbone followed by formation of prenylated chemically self-assembled nanorings.

**Figure 7:**
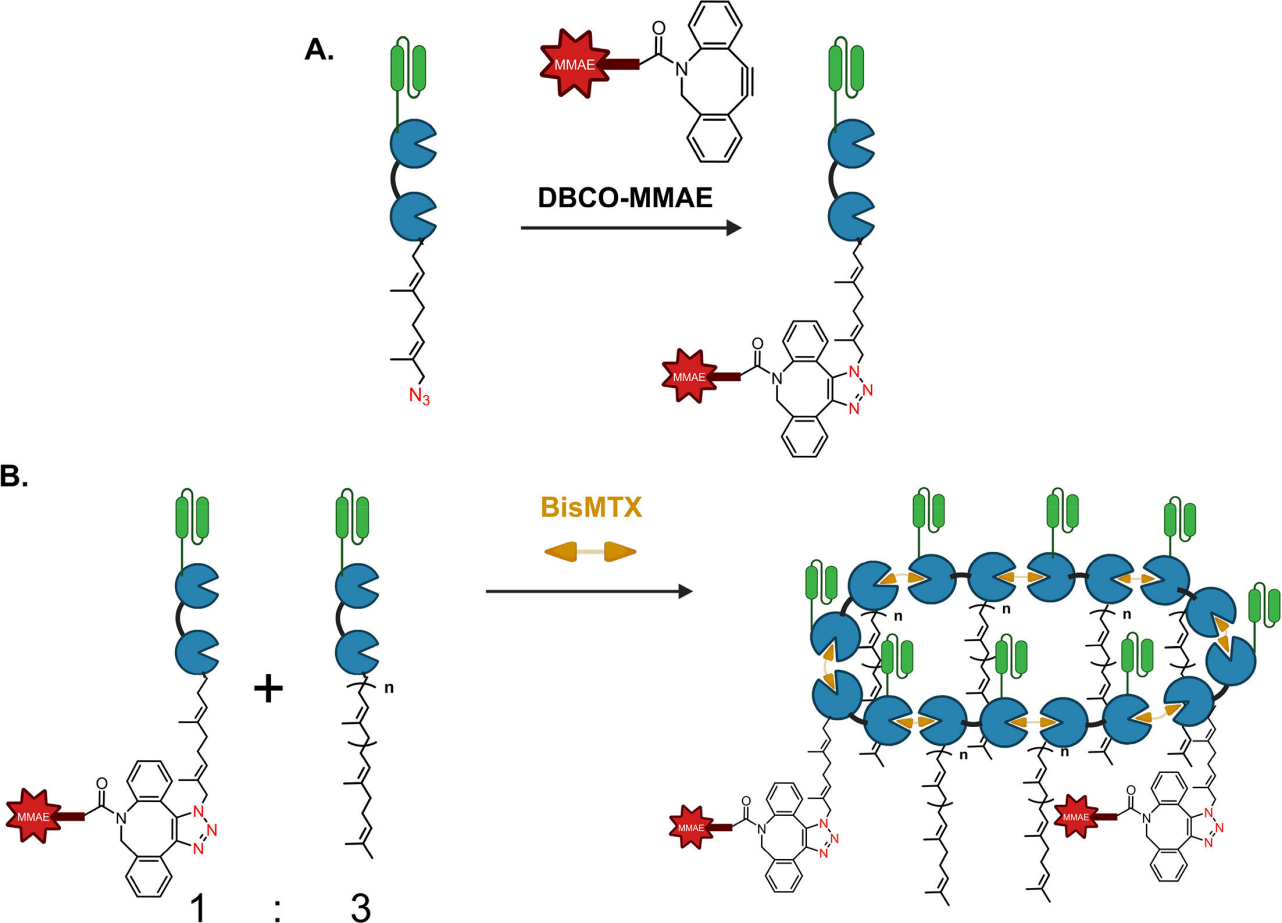
Assembly of MMAE-CSANs. (**A**) DHFR^2^ monomer modified with **C10-Azi** followed by click reaction performed with DBCO-MMAE. (**B**) MMAE-CSANs formed by combining the MMAE-functionalized monomer with the farnesylated monomer in a 1:3 ratio. In these CSANs, farnesyl (*n* = 1) or geranylgeranyl (*n* = 2) groups can be used.CSANs, chemically self-assembled nanorings; MMAE, monomethyl auristatin E.

**Figure 8:**
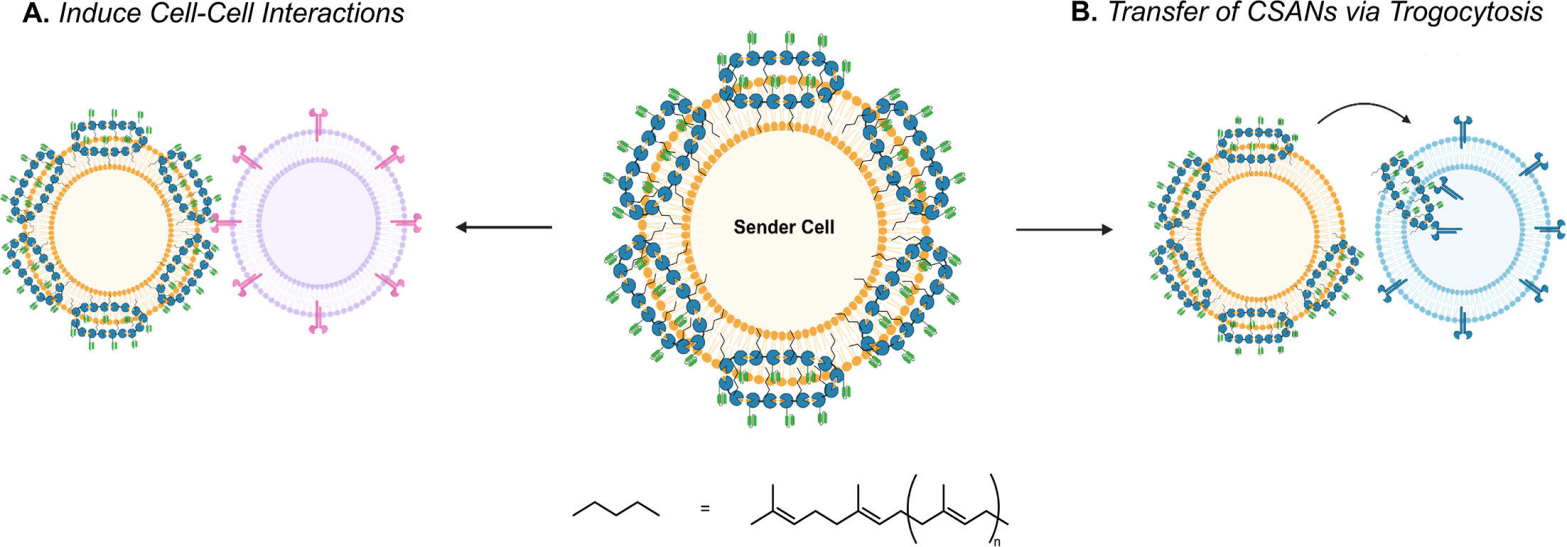
Cell surface modification using prenylated CSANs. (**A**) Induction of cell–cell interactions. (**B**) CSANs transfer entirely to target cells. In these CSANs, farnesyl (*n* = 1) or geranylgeranyl (*n* = 2) groups can be used. CSANs, chemically self-assembled nanorings.

Protein-based nanorings have shown utility in drug delivery and inducing cell–cell interactions. These nanorings are multivalent, stable on cell surfaces, and can be appended with drugs or other chemical entities [134,135]. They are composed of a protein backbone consisting of two dihydrofolate reductase proteins genetically fused together with a glycine linker (DHFR^2^) [134–140]. In the context of prenylation, the DHFR^2^ backbone has been engineered to contain a C-terminal CaaX sequence that allows the protein to be enzymatically prenylated, either with farnesyl or geranylgeranyl groups (Figure 6) [141]. This modification functionalizes the nanorings for broad application either by themselves or on cell surfaces (Figures 7 and 8) [134,135,141]. These prenylated proteins can then be induced to undergo chemically self-assembled nanoring (CSAN) formation upon addition of a chemical dimerizer, bis-methotrexate (BisMTX, Figure 6), resulting in the formation of primarily octameric rings, 33–34 nm in diameter. The DHFR^2^ component of nanorings can be fused with targeting proteins, such as single-chain fragment variants (scFvs) or fibronectin domains to target cell surface receptors specific to cancer or other cell types. The resulting rings are multivalent, targetable, and can be further functionalized with drugs or nucleic acids.

Prenylated CSANs have been shown to be stable and broadly applicable for studying and exploiting cell-cell interactions. These nanorings have been dual labeled with TAMRA and GFP fluorophores using click chemistry and farnesylation [128]. This dual fluorophore labeling enabled CD3 internalization into T-leukemia cells to be studied. After a 24 hour incubation at 37°C, CD3 internalization, based on fluorescence microscopy and FRET, was observed. Thus, this dual protein labeling strategy allowed for internalization studies as well as FRET observation.

CSANs with a prenyl group have also been used for drug delivery. Azide analog, **C10-Azi**, was appended onto the DHFR^2^ protein scaffold using FTase and further functionalized via SPAAC modification of the CSANs with DBCO-containing drugs or nucleic acids. An anti-EGFR fibronectin domain was also fused to the DHFR^2^ element in the CSANs to target e.g.FR positive cell lines. Wang and colleagues investigated the ability of DBCO-MMAE functionalized CSANs to induce specific toxicity based on elevated EGFR receptor expression in cancer cells[142]. Using cell lines with variable EGFR expression levels including A431 (high), MDA-MB-231 (medium high), MCF-7 (medium low), and MDA-MB-453 (low), they determined EC_50_ values for the nanoring-drug conjugates. Similar EC_50_ values were observed for DBCO-MMAE-CSANs compared with free MMAE drug in cell lines with high EGFR expression, indicating general cytotoxicity. In contrast, EC_50_ values for MMAE-linked CSANs increased in cell lines with lower expression (compare 2.4 nM in A431 cells versus 41 nM in MDA-MB-453 cells), demonstrating selective toxicity for cells with high levels of EGFR expression. Importantly, free drug MMAE showed minimal differences in EC_50_ values across cell lines, and cytotoxicity was not correlated with receptor expression. Demonstrating the selectivity of this approach highlights the importance of valency for surface receptors in tuning specific protein chemotherapies.

An important feature of the CSAN design is that the pendant prenyl groups allow for modification of any cell surface, without the need for re-engineering the DHFR^2^ protein scaffold. Prenylated CSANs can be incubated with various cell types and have been shown to remain bound for >72 hours [141]. Decoration of cells with prenylated CSANs can be used to induce cell–cell interactions or deliver drugs and biomolecules through transfer of CSANs from the surface of sender cells (cells initially treated with CSANs) to receiver cell surface via trogocytosis[142,143]. Wang et al. demonstrated that prenylated CSANs can induce reversible cell-cell interactions between peripheral blood monocytes and EGFR-positive cancer cells. Additionally, when click chemistry was performed using the pendant azide handles, drugs and nucleic acids could be delivered to receiver cells to achieve potent cytotoxic effects. These studies highlight the broad applicability of prenylation for use in cell-based therapies, without the need for genetic modification.

## Multivalent protein–PEG conjugates

**Figure 9:**
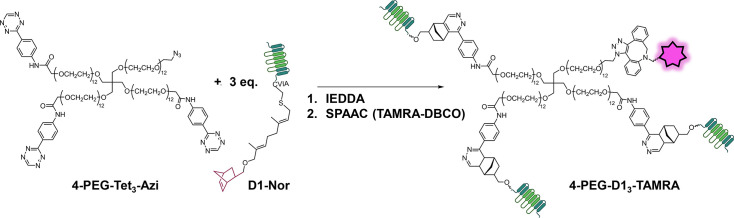
Schematic representation of 4-PEG-D1_3_-TAMRA synthesized via one-pot norbornene-tetrazine ligation and SPAAC. The D1 protein is shown in green and TAMRA fluorophore as a pink star. SPAAC: strain-promoted azide alkyne cycloaddition.

As noted above, **C10-Nor** was shown to undergo IEDDA reactions with tetrazine-containing reagents, enabling copper-free protein labeling. Building on this, the orthogonality of IEDDA with respect to SPAAC was leveraged to develop a multiprotein–polymer assembly using a combination of norbornene–tetrazine conjugation and SPAAC. Those reactivity differences highlighted the utility of dual orthogonal reactions for the efficient assembly of protein conjugates with novel structures and functions. A trimeric construct using a four-arm PEG polymer core linked to three EpCAM-targeting DARPin proteins and a TAMRA fluorophore was designed (4-PEG-D1_3_-TAMRA, Figure 9). Affinity titration experiments with EpCAM^+^ MCF7 cells yielded similar *K*_*d*_ values for both the trimeric and monomeric (D1-TAMRA) constructs (7.6 nM and 3.8 nM, respectively). However, the monomer, but not the trimer, exhibited substantial variability at high protein concentrations, suggesting that the PEG polymer core was conferring a stabilizing effect on the lipid-modified DARPins. The absence of improved affinity due to multivalent binding with this construct likely stems from the short PEG arms (12 units), which may be too short to effectively bridge the distance between adjacent EpCAM molecules. This modular design could be optimized for multivalent interactions by extending the PEG arms or targeting more densely packed cell surface proteins. Notably, PEGylation improved protein handling during FTase-catalyzed modification, mitigating solubility issues sometimes observed with hydrophobic isoprenoid-modified proteins. This strategy, leveraging two distinct bioorthogonal reactions, offers a versatile platform for assembling a variety of multifunctional protein conjugates [50].

## Modulating substrate specificity via mutagenesis

In addition to the chemical approaches described in the above sections, site-directed mutagenesis has been used to modulate and improve the properties of FTase for biotechnology applications. Mutagenesis and peptide selectivity studies revealed that FTase specificity is governed by multiple interactions and can be tuned through manipulation of key active site contacts. For the a_2_ position of the CaaX sequence (Ca_1_a_2_X), both the steric volume and polarity of the residue play a crucial role in substrate discrimination [144]. The a_2_ binding site is composed of Trp102β, Trp106β, Tyr361β residues and the third isoprenoid unit of the FPP, presenting a hydrophobic pocket that favors moderately sized nonpolar amino acids and excludes large or charged residues. Substituting Trp102β and Trp106β with smaller (Ala, Val) or polar (His) residues altered FTase specificity significantly [145]. Reducing steric bulk from Trp to Val or Ala increased reactivity with a CVWS substrate up to 25-fold. The W102Hβ and W106Hβ mutations enhanced *k*_cat_/*K*_*M*_ for CVDS farnesylation by ~10- and 4-fold, respectively, and the double mutant (W102Hβ/W106Hβ) showed a ~30-fold increase in *k*_cat_/*K*_*M*_ compared with wildtype (WT) FTase. Remarkably, screening of the W102/W106 mutant library identified variants up to 10^4^-fold increased reactivity with Ca_1_a_2_X substrates containing Lys or Asp at a_2_, demonstrating catalytic efficiencies comparable with WT FTase. This enhanced reactivity arises from two key factors. First, charge complementarity between the mutant enzyme and the a_2_ residue of the target Ca_1_a_2_X motif, where Arg or Lys is present in variants active with CVDS, while Glu or Asp is found in those reacting with CVKS. Second, the active site pocket is expanded through the incorporation of smaller residues, allowing for improved substrate accommodation. These mutations allow charge matching while maintaining FTase’s reactivity with native substrates, indicating that Trp-102β and Trp-106β primarily restrict substrate flexibility rather than enhancing catalytic activity. The combination of positive interactions, which stabilize substrate binding, and negative interactions, which discriminate against non-substrates, allows FTase to maintain a broad yet selective substrate range, fulfilling its functional requirement for multispecificity [145].

In a related study, mutation of W102β to Thr and Y365β to Phe enabled FTase to utilize GGPP as a substrate (GGPP is normally an inhibitor [36]), with single mutant, W102Tβ, acting as a molecular switch for isoprenoid selectivity [146,147]. A major limitation in the use of non-natural FPP analogs with bioorthogonal functionality as substrates for FTase is their reduced activity, particularly for bulkier derivatives. Studies have shown that mutating key residues that interact with the third isoprene unit—W102β, Y154β, and Y205β—to Thr enhanced the incorporation of biotin [148] and nitrobenzofurazan (NBD) containing FPP analogs [149]. Similarly, mutations of these residues to Ala improved reactivity with benzaldehyde-, cyclooctene-, NBD-, and coumarin-containing FPP analogs. A W102Aβ mutation resulted in a four-fold increase in *k*_cat_ and a 10-fold decrease in *K*_*M*_ for **C10-BA**, leading to a 40-fold overall gain in catalytic efficiency. Likewise, the Y205Aβ mutation enhanced efficiency by 300-fold due to a 25-fold increase in *k*_cat_ and a 10-fold decrease in *K*_*M*_ for a coumarin-containing analog, **C10-Cou** (Figure 4). Interestingly, the Y205A mutant displayed a higher *K*_*M*_ than the wild-type enzyme for the **C10-TCO**, yet its *k*_cat_ was approximately five-fold greater. Since the Y205Aβ mutant exhibited a submicromolar *K*_*M*_, the enzyme can be readily saturated with **C10-TCO** and hence fully capitalize on the five-fold increase in *k*_cat_ [150]. Enhanced catalytic activity, driven by increased *k*_cat_, is particularly useful for FTase-based labeling *in vitro* to increase product throughput. For *in vivo* applications, where intracellular FPP analog concentrations are likely to be low, reduced *K*_*M*_ parameters are equally important for efficient substrate labeling. Overall, FTase mutagenesis has expanded the scope of incorporation of a diverse range of substrates of different steric volumes for a plethora of applications.

## Conclusions

**Figure 10:**
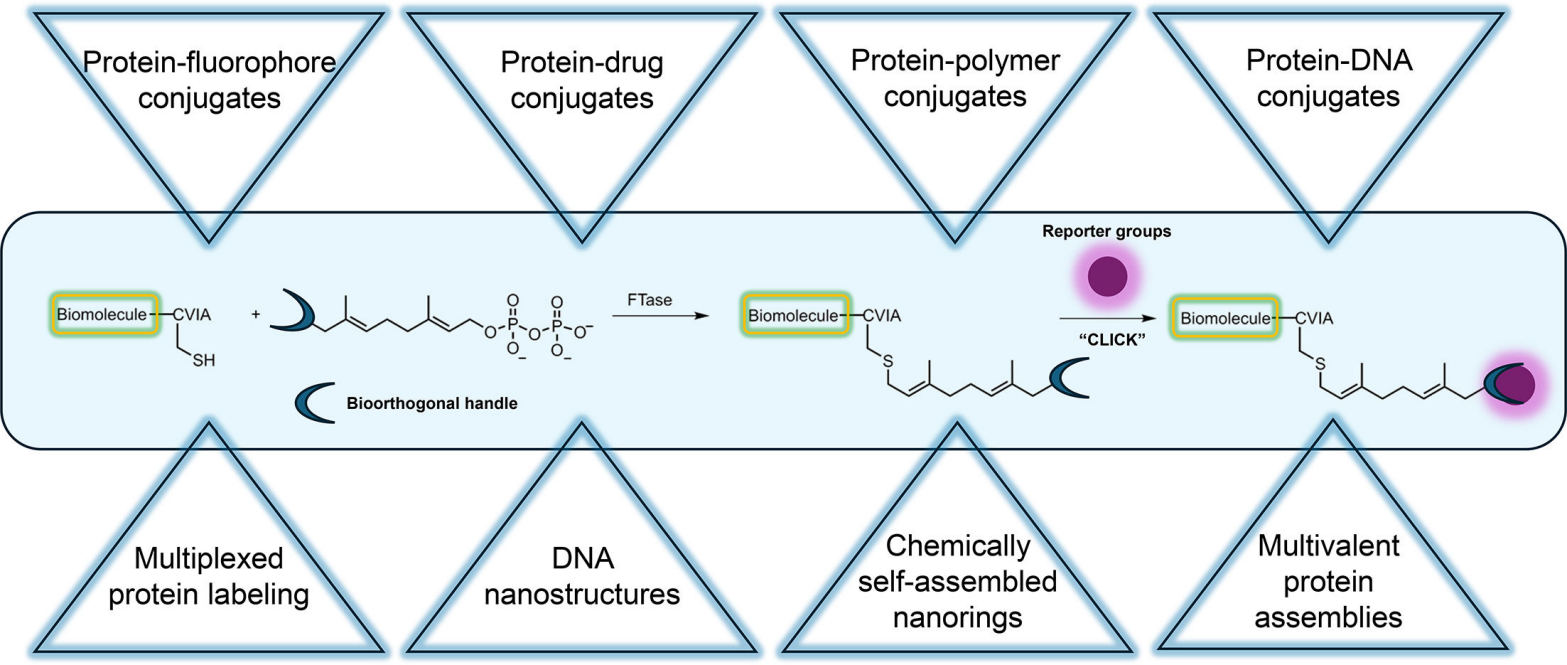
Schematic overview of FTase-mediated chemoenzymatic site-specific protein labeling, illustrating its broad utility across biotechnological and therapeutic applications. FTase: farnesyltransferase.

A wealth of research on FTase has led to the development of robust methods for chemoenzymatic site-specific protein modification, opening new avenues for biotechnology applications and therapeutic development (Figure 10). The versatility of FTase enables the incorporation of a broad range of bioorthogonal handles at the C-terminus of proteins, while typically maintaining their native functionality. Furthermore, the precision of bioorthogonal chemistry facilitates the selective modification of target proteins, both in isolated forms and within complex protein mixtures. In addition, FTase-mediated protein modification has enabled the construction of multivalent protein assemblies, while the hydrophobic lipid tail facilitates membrane integration, thereby enhancing cell–cell interactions and the delivery of therapeutic cargo to target cells. Mutagenesis studies clearly demonstrate that FTase can be tuned to accommodate larger substrates and non-native CaaX-box sequences. Collectively, these advances should pave the way for the development of more sophisticated protein modifications and advanced biomolecular systems, with enhanced functionality.

**Perspectives**

- Farnesyl transferase (FTase)-protein substrate recognition is primarily dictated by the identity of four residues positioned at the C-terminus. This allows virtually any protein to be converted into an FTase substrate, providing a versatile catalytic approach for bioconjugation and precise protein functionalization.
- The intrinsic substrate tolerance of FTase allows site-specific incorporation of diverse bioorthogonal handles, expanding its utility in drug delivery, imaging, and biomaterials.
- Expanding the FTase active site to accommodate bulkier or more hydrophilic isoprenoid analogs could significantly broaden its utility. Future studies should focus on leveraging FTase-mediated modifications in vivo to enhance their impact in biotechnology. CRISPR/Cas9 offers the ability to precisely insert CaaX motifs into endogenous proteins, enabling site-specific FTase labeling while preserving physiological expression. Synthetic biology tools will provide spatial and temporal control over prenylation, allowing targeted protein labeling in specific cell types or conditions. When coupled with bioorthogonal chemistry, this platform holds strong potential for real-time imaging and therapeutic modulation of proteins within living systems.

## Abbreviations

ADC: antibody–drug conjugates
Bis-MTX: bis-methotrexate
CSANs: chemically self-assembled nanorings
CuAAC: copper-catalyzed azide alkyne cycloaddition
DARPins: designed ankyrin repeat proteins
DBCO: dibenzocyclooctyne
DHFR: dihydrofolate reductase
DPC: DNA-protein cross-links
EGFR: epidermal growth factor receptor
EpCAM: epithelial cell adhesion molecule
FPP: farnesyl diphosphate
FRET: Förster resonance energy transfer
FTase: farnesyltransferase
GGPP: geranylgeranyl diphosphate
GIP: glucose-dependent insulinotropic polypeptide
GSTase: glutathione S-transferase
Hcp1: homohexameric hemolysin-coregulated protein
I: geranylgeranyltransferase type I
IEDDA: inverse electron demand Diels–Alder reaction
II: geranylgeranyltransferase type II
MMAF: monomethyl auristatin F
NBD: nitrobenzoxadiazole
PDC: protein–drug conjugates
PEG: polyethylene glycol
SPAAC: strain-promoted azide-alkyne cycloaddition
TCO: trans-cyclooctene
Tz: tetrazine
eGFP: enhanced green fluorescent protein
m/p-PDA: meta/para-phenylene diamine
protein-ODN: protein–oligonucleotide conjugates
scFvs: single-chain fragment variants
